# Moms and Mercury: Fine-Tuning Fish Consumption During Pregnancy

**Published:** 2005-10

**Authors:** Ernie Hood

Due to ongoing concerns that high mercury intake via fish can cause adverse neurologic effects in the developing fetus, the U.S. Food and Drug Administration now recommends that expectant mothers should limit their consumption of fish to two or fewer meals per week. But pregnant women shouldn’t throw the baby out with the bathwater. A new study by a group of Harvard researchers suggests that this advice, which could result in many pregnant women eliminating fish from their diets altogether, may be denying some babies substantial neurocognitive benefits gained from important nutrients found in fish, such as n-3 polyunsaturated fatty acids **[*EHP* 113:1376–1380]**.

The scientists sought to determine whether fish consumption during pregnancy is harmful or beneficial to fetal brain development. To do this, they examined associations of maternal fish consumption during pregnancy, maternal hair mercury levels (a sensitive marker of organic mercury body burden) at delivery, and infant cognition at age 6 months. Study subjects were 135 mother–infant pairs who participated in Project Viva, a prospective pregnancy and child health cohort study in eastern Massachusetts.

The mothers completed questionnaires about fish consumption during their second trimester. That period of time was used to best coordinate temporally with the mercury exposure reflected in maternal hair samples, which were taken at delivery. The questions concerned how much and what categories of fish (canned tuna, dark meat, light meat, shellfish) the women ate.

Mothers consumed an average of 1.2 servings of combined fish categories per week. Their mean hair mercury level was 0.55 part per million (ppm), with 10% of the samples higher than 1.2 ppm, the current U.S. reference dose. Fish consumption was directly correlated with hair mercury levels.

Infant cognition was assessed using a test called visual recognition memory (VRM). In the VRM test, which has been shown to correlate with later IQ, the child is first shown two identical photographs of an infant’s face, side by side, at a standardized distance. Then, one of the photos is replaced with a photo of another infant’s face. By tracking the percentage of time the baby looks at each photo, a novelty preference score is derived, reflecting the infant’s ability to encode a stimulus into memory, to recognize that stimulus, and to look preferentially at a novel stimulus.

Mean VRM score among the children was 59.8, with a range of 10.9–92.5. After accounting for characteristics such as maternal age and education level, higher fish intake was found to be associated with higher infant cognition, especially after adjusting for mercury levels, which had a dose-dependent negative impact on the infants’ cognition. For each additional weekly serving of fish, the infants’ VRM score was 4.0 points higher. Conversely, the researchers found that an increase of 1 ppm in hair mercury was associated with a decrement in VRM score of 7.5 points. The babies with the highest cognition scores were from mothers who had eaten more than two weekly fish servings but had mercury levels of 1.2 ppm or less.

Although the results may seem contradictory, the authors suggest that the most cognitive benefit is derived by mothers eating fish types with the combination of relatively little mercury and high amounts of beneficial nutrients. However, since the study assessed maternal fish consumption of four broad categories, there is no information presented on associations with specific types of fish. The researchers say that future studies could incorporate more detailed dietary information to help pregnant women make informed decisions about which fish meals are better or worse for their children’s cognition.

Ultimately, the message behind these findings is that pregnant women should continue to eat fish, but should try to choose varieties known to be low in mercury and high in nutrients, such as canned light tuna and sardines. Finding the most appropriate balance between risk and benefit may be challenging in this situation, but given the strong associations found in the current study, making the right decisions about which fish to eat during pregnancy, and how often, may be even more important than previously suspected.

## Figures and Tables

**Figure:**
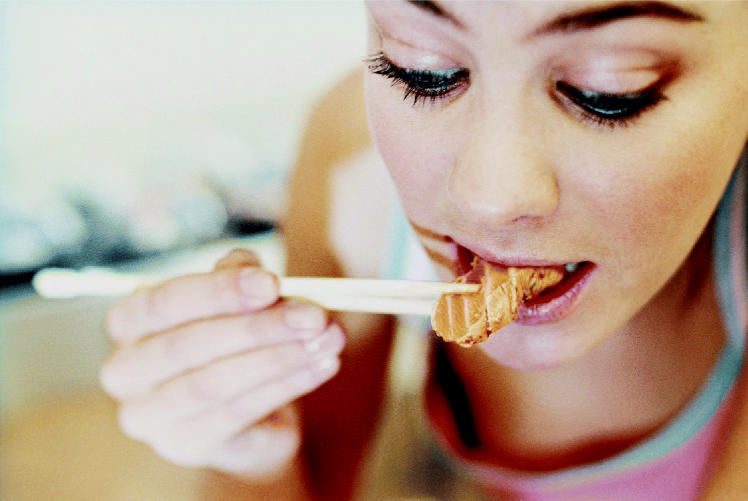
Eating for two, thoughtfully. Despite the threat posed by high mercury levels in certain types of fish, new findings suggest a healthy prenatal diet most likely should include some low-mercury seafood.

